# Can diffusion tensor imaging unlock the secrets of the growth plate?

**DOI:** 10.1093/bjro/tzae005

**Published:** 2024-02-15

**Authors:** Ola Kvist, Laura A Santos, Francesca De Luca, Diego Jaramillo

**Affiliations:** Department of Paediatric Radiology, Karolinska University Hospital, Stockholm, 171 64, Sweden; Department of Women’s and Children’s Health, Karolinska Institute, Stockholm, 171 77, Sweden; Department of Radiology, Columbia University Irvine Medical Center, New York, NY 100 32, United States; Department of Radiology, Karolinska University Hospital, Stockholm, 171 64, Sweden; Department of Clinical Neuroscience, Karolinska University Hospital, Stockholm, 171 65, Sweden; Department of Radiology, Columbia University Irvine Medical Center, New York, NY 100 32, United States

**Keywords:** growth plate, diffusion tensor imaging, growth, tractography

## Abstract

“How tall will I be?” Every paediatrician has been asked this during their career. The growth plate is the main site of longitudinal growth of the long bones. The chondrocytes in the growth plate have a columnar pattern detectable by diffusion tensor imaging (DTI). DTI shows the diffusion of water in a tissue and whether it is iso- or anisotropic. By detecting direction and magnitude of diffusion, DTI gives information about the microstructure of the tissue. DTI metrics include tract volume, length, and number, fractional anisotropy (FA), and mean diffusivity. DTI metrics, particularly tract volume, provide quantitative data regarding skeletal growth and, in conjunction with the fractional anisotropy, be used to determine whether a growth plate is normal. Tractography is a visual display of the diffusion, depicting its direction and amplitude. Tractography gives a more qualitative visualization of cellular orientation in a tissue and reflects the activity in the growth plate. These two components of DTI can be used to assess the growth plate without ionizing radiation or pain. Further refinements in DTI will improve prediction of post-imaging growth and growth plate closure, and assessment of the positive and negative effect of treatments like cis-retinoic acid and growth hormone administration.

Diffusion tensor imaging (DTI) was developed from diffusion weighted imaging (DWI) in neuroradiology to gain additional information about the brain and its microstructure.^1^ A large focus has been to visualize the neuronal pathways in the central nervous system to investigate how various areas of the brain are being interconnected with each other. DTI has later expanded its application from the central to the peripheral nervous system, and, more recently, has been used in abdominal radiology to identify early signs of organ transplant rejection, as well as in musculoskeletal radiology.^1^

The locomotor system is very well suited for MRI and DTI. The symmetrical organization of cells in muscle fibres, and of the chondrocytes in the growth plate, is conducive to imaging with DTI. This review article will illustrate how DTI can be used in the paediatric locomotor system and provide future perspectives in this field of research.

## Growth plate

The growth plate is a cartilaginous structure located near the ends of the long bones and responsible for longitudinal growth. The chondrocytes in the growth plate are divided into three layers with specific characteristics. The chondrocytes originate from the resting or germinal zone to proliferate where they start to be arranged in a columnar pattern (proliferate zone) until they increase in size (hypertrophic zone) and excrete extracellular matrix as part of osteogenesis. Both the chondrocytes in the proliferate and hypertrophic zones as well as the newly formed bone in the metaphysis have a symmetrical columnar pattern.^2^ The columnar pattern creates an anisotropic diffusion in the growth plate, especially in the zone of provisional calcification in the hypertrophic zone. DTI is a method to evaluate if the diffusion (Brownian motion of water) is equal in every direction (isotropic) or if the diffusion is more pronounced in one direction (anisotropic) in a tissue.^1^ The directional diffusion is measured as fractional anisotropy (FA), which is a scalar value ranging between 0 and 1 where 0 is isotropic diffusion and 1 is completely directional anisotropy. Other aspects of diffusion can be measured with DTI such as mean diffusivity (MD), axial diffusivity (AD), and radial diffusivity (RD).^3^ The average the three eigenvalues (λ1, λ2, and λ3) of the diffusion tensor is called MD, roughly equivalent to the apparent diffusion coefficient. The largest eigenvalue is the axial diffusivity (AD) and the average of the two smaller eigenvalues is the radial diffusivity (RD).^1^

**Figure 1.**
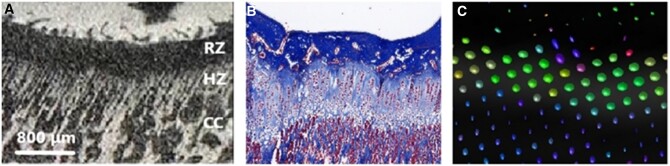
Comparison of μCT, histology and DTI at the growth plate in a rabbit study. (A) Close-up of the growth plate on μCT (10 μm voxel size) rabbit shows a brush like appearance of bone trabeculae columns in the hypertrophic zone and its transition to the metaphysis. (B) Histology sample with Masson trichrome staining shows columns of enlarged chondrocytes in the same region. (C) Close-up of the corresponding FA map to the histology sample shows mostly a lateral diffusion in the central portion of the growth plate but more perpendicular in the transitional zone between the growth plate and the metaphysis. The growth velocity can be considered as a product of the production rate of chondrocytes in the proliferative zone and the vertical height of chondrocytes in the hypertrophic zone. Courtesy of Kvist et al.^10^

The columnar pattern creates anisotropic diffusion which has been evaluated in numerous studies.^4–12^ The relationship between anisotropy, columnar chondrocytes and the calcified cartilage in the hypertrophic zone has been confirmed in a rabbit study comparing DTI, μCT, and histology (Figure 1).^10^

## Acquisition

DTI-MRI of the knee is performed with a 3 T whole-body scanner. A fat-suppressed single shot spin echo echo-planar sequence *b-*values of 0 and 600 s/mm^2^. The number of directions and other specific imaging parameters are slightly different depending on the vendor and can be seen in Table 1.^8^^,^^11^^,^^13^

**Table 1.** Technical settings for DTI for each vendor.

Technical settings	GE^13^	Siemens^8^	Philips^11^
Number of directions	20	20	15
Repetition time (TR)/echo time (TE)	3000/51.7 ms	7100/82 ms	7100/80 ms
Bandwidth	1953.12 Hz/pixel	1446 Hz/pixel	40 Hz/pixel
Parallel imaging factor	2	2	2
Signal averages	5	2	2
Matrix size	255 × 255	128 × 128	54 × 54
Field of view	256 × 256	256 × 256 mm	162 × 162
Voxel dimensions	2 × 2 mm	2 × 2 mm	3 × 3 mm
Slice thickness	3 mm	3 mm	3 mm
Gap between slices	No gap	No gap	No gap

## Post-processing

**Figure 2.**
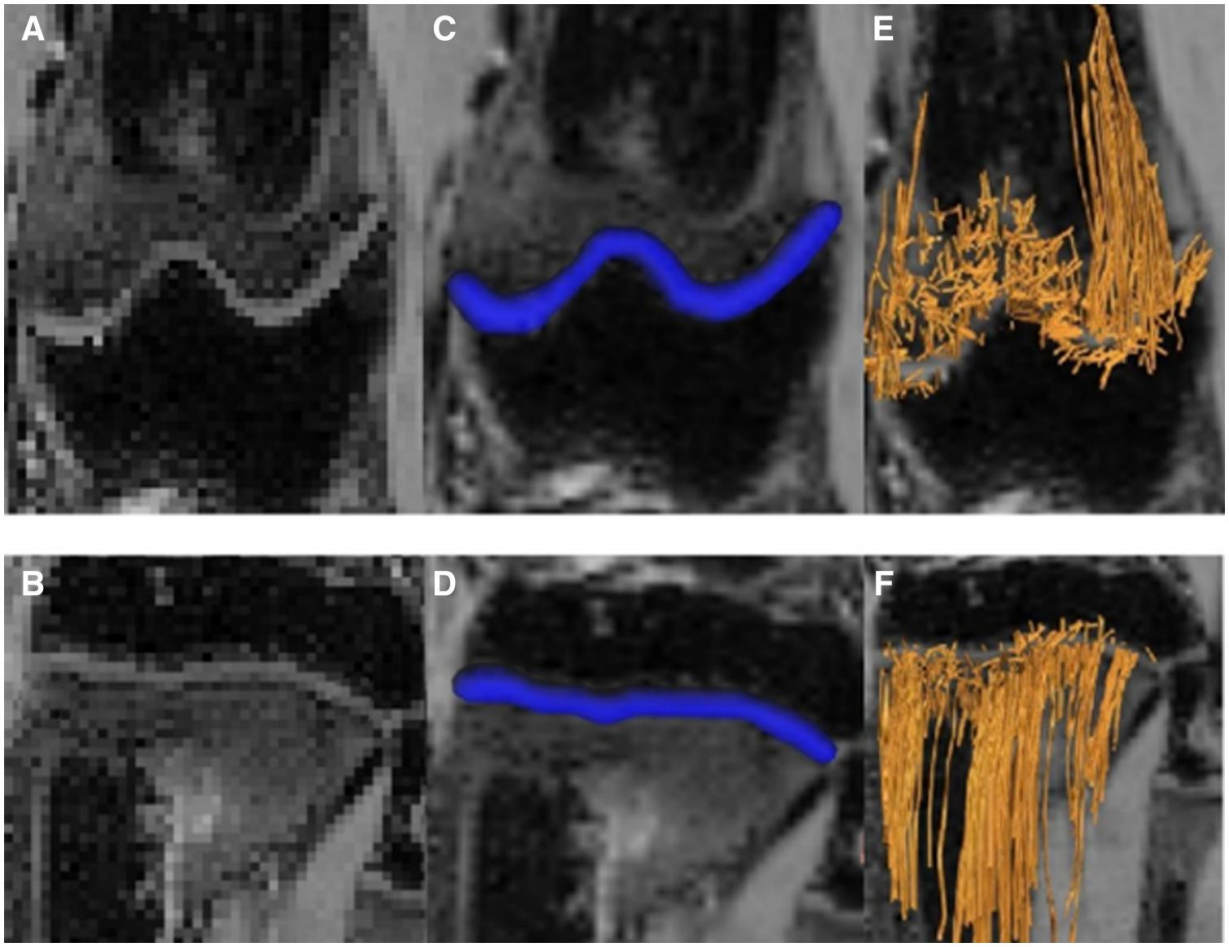
Image segmentation. The growth plate is segmented manually on a coronal b0 image in both femur (A) and tibia (B). The traced ROI is seen as blue (C, D). From the ROI can tractography be performed when maximum angular turning angle and minimum FA threshold has been added (E, F). Courtesy of Kvist et al.^10^

Additional information can be extracted from DTI by post-processing tractography. The post-processing is currently done manually by tracing the growth plate in each image to create a region of interest (ROI). Processing of the DTI ROIs provides the quantitative growth plate information and the tractography images (Figure 2). Tractography, developed to create a 3D model to analyse neuronal pathways in the brain, is a post-processing technique which creates a fibre by analysing the diffusion tensor voxel-by-voxel with maximum turning angle and a minimum FA value threshold. The most common tractography technique is anatomical ROI-seeding, ie, the ROI is the starting point, and the tract will continue if it fulfils the maximum turning angle and minimum FA value requirement. Another technique uses two ROI’s, with thresholds of maximum turning angle and minimum FA value on a voxel-by-voxel basis between the two ROI’s. Previous experimental DTI studies have shown that this method is feasible, and the results are reproducible.^7^^,^^10^

Tractography is also dependent on the technical parameters, especially the number of directions the diffusion is measured. It has been reported that the results from the tractography were stable when the number of directions was more than 18. However, a follow-up study stated that the tract length and volume increased significantly when the number of angles was less than 40 and that the fractional anisotropy was stabilized at 14 directions.^14^ On the contrary, the MD value was not affected substantially by the number of directions measured.

The b-value affects both the tractography and the DTI metrics of the growth plate. The tract volume increases with higher b-values whereas tract length, FA and MD value reach a plateau with a b-value higher than 750 s/mm^2^.^15^ To obtain these b-values, given that higher b-values lead to decreased SNR, the imaging time needs to be increased to maintain image quality.

Animal studies suggest that DTI of the growth plate require a b-value between 500 and 1500 s/mm^2^, SNR higher than 10 and more than 14 directions. To make this feasible in a paediatric population we have used a 3 T whole-body scanner, approximately 20 noncollinear directions and b-values of 0 and 600 sec/mm^2^.

## Velocity

It is common knowledge that females mature earlier than males, most famously presented by Greulich and Pyle (GP).^16^ The growth activity, and thus, the growth velocity can be measured by the turnover rate of the chondrocytes in the proliferative zone and their enlargement in the hypertrophic zone.^17^ This in turn suggested that the growth could be measured by multiplying the number of chondrocytes in the proliferative zone with the height of the chondrocytes in the hypertrophic zone (G = N*hmax).^17^ The transitional zone between growth plate and bone has a symmetrical pattern with calcified cartilage creating palisades that should be measurable by DTI. A major concern has revolved around whether DTI reflects the growth plate and its activity or not. The study by Kvist et al has shown that DTI can measure the active part of the growth plate and Zhao et al and Wang et al have proposed optimal b-value and angular resolution for DTI in the knee joint.^10^^,^^14^^,^^15^ A comparison between high-resolution µCT and DTI showed that the calcified cartilage in the hypertrophic zone and primary spongiosa has a comb-like distribution which correlates with the DTI findings (Figure 1).

The main question in is whether we can implement these findings in children and adolescents and see the activity of the growth plate, ie, growth velocity. Sander multiplier, White-Menelaus predictor and Paley multiplier have stated that there is an annual longitudinal growth of 0.95 cm/year for the distal femur and 0.64 cm/year for the proximal tibia in 10-15-year-olds.^18^ A retrospective DTI study with 89 participants, aged between 4 and 17 years of age, showed that the tract volume had the greatest correlation with growth velocity and total height gain.^9^ Thus, DTI can function as a biomarker for early detection of increased or decreased growth velocity.

**Figure 3.**
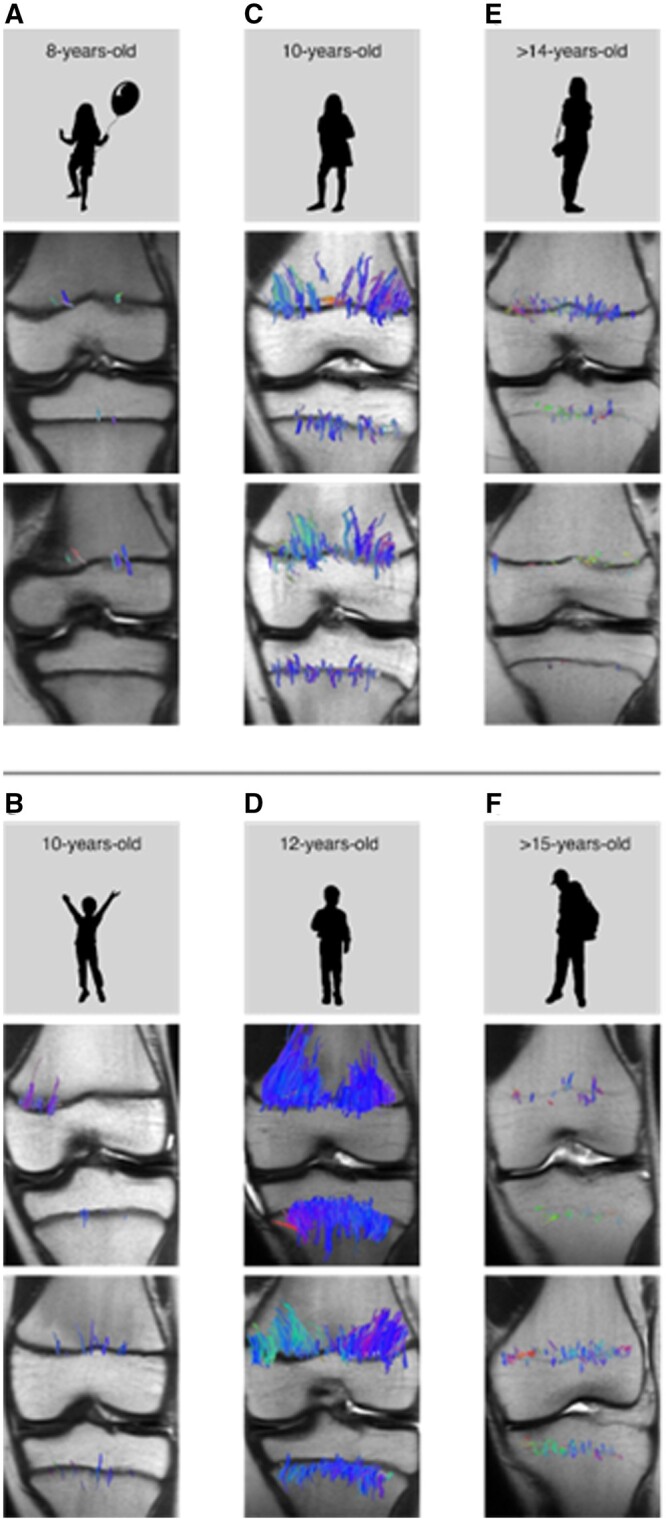
Tractography superimposed on T1-weighted images in children transitioning into puberty: 8-year-old females (A) and 10-year-old males (B). Femoral and tibial fibre tracts are scant, short, and parallel to the long axis of the bone in both females and males. In children experiencing peak growth, the physeal tractography pattern increases in density and thickness because these children are undergoing rapid bone growth. Rapid cell proliferation and hypertrophy results in both increased chondrocyte column height and increased stature. In this phase the columns are longer, more densely packed, and organized in parallel, characteristics that are reflected in the tractography pattern (C, D). After the growth spurt the tracts becomes less dense, shorter and more asymmetrical (E, F).

**Figure 4.**
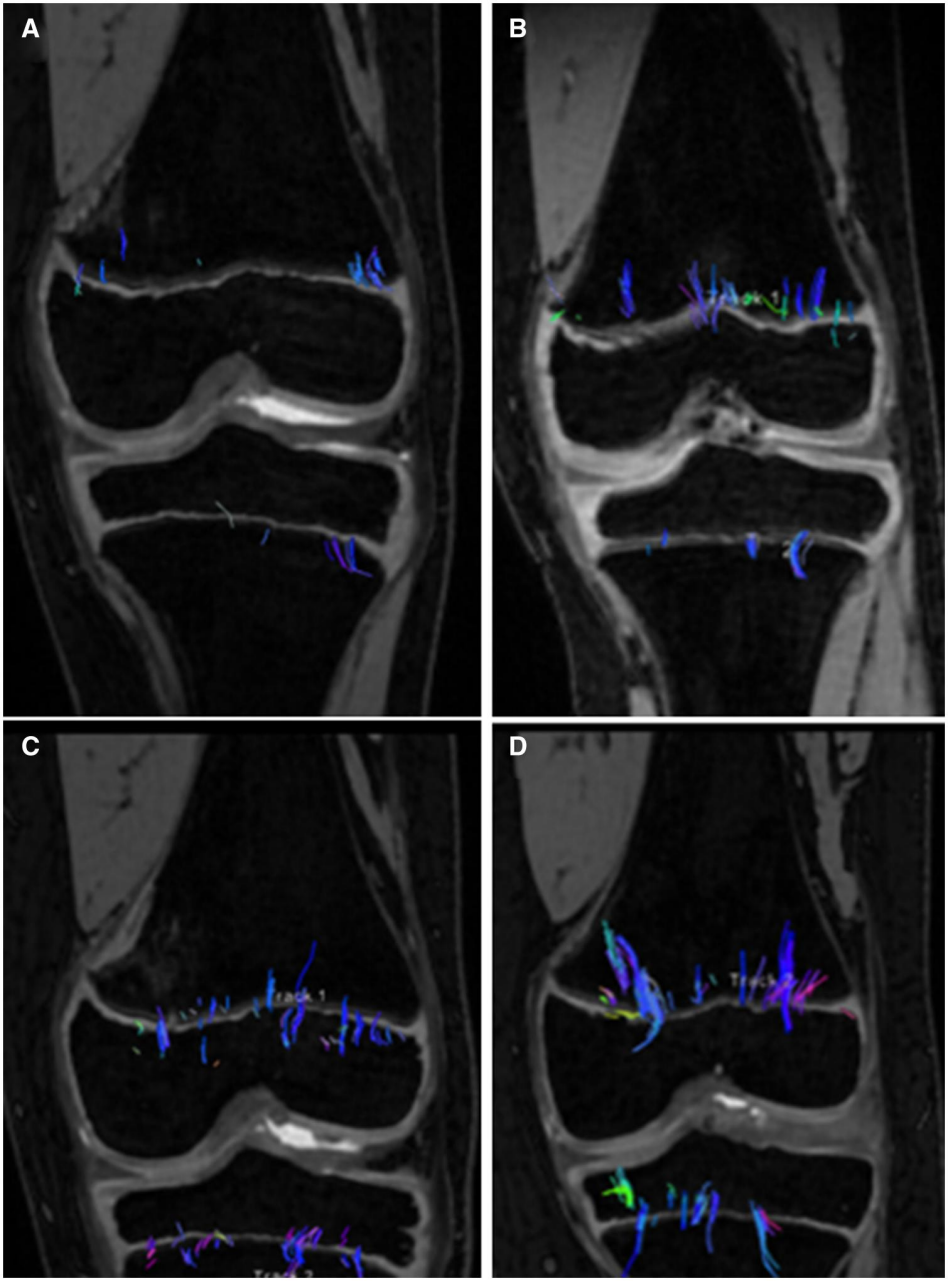
Diffusion tensor imaging fibre tract patterns will be reflective of skeletal age, with little to no correlation to chronological age. In the figure (A, B) are fibre tracts in two females with a bone age of 9 years old. Furthermore, A is chronologically 10-years-old and B is chronologically 8. (C, D) are fibre tracts for two females with a 9-year-old bone age, however, (C, D) both have a 10 year-old chronological age. There is an evident increase in fibre tract number and density in females with a bone age of 9 years old compared to females who are skeletally 8 years old in which very little, scant, and thin fibres are seen.

Tractography has been documented to have a correlation with growth velocity. The tibial and femoral tracts begin to lengthen about the age of 8 in girls and the age of 10 in boys (Figure 3).^7^ The distribution of tracts varies with most of the tracts in the peripheral portions of the femoral growth plate while the tracts in the tibia are more centrally located. The peak of longitudinal growth is indicated by an increase of number of tracts and their volume. This is a result of the increased length of cartilage columns previously discussed which is characterized on tractography by long, densely packed tracts that are parallel to each other and perpendicular to the growth plate.^5^ This relationship has been proven to be related to the skeletal maturity of the growth plate and not the chronological age of an individual (Figure 4).^11^ After the growth spurt, the tracts become thinner, shorter, and increasingly disorganized, related to the beginning of the growth plate senescence. At the end-stage of senescence, only very few, disorganized tracts are apparent. In the skeletally immature child, there is higher physeal cartilage composition due to unmineralized chondrocyte columns in the physes and adjacent metaphyses. MR-DTI depicts physeal microstructure through diffusion of water molecules. The measurement of Signal-to-Noise Ratio (SNR) in both open and closed physes has consistently shown higher SNR in open physes.^7^^,^^8^ Bedoya et al and Jaimes et al conducted DTI-MR SNR measurements on a cohort of over 180 children, ages 8-18 years old, revealing decreasing SNR values in children approaching skeletal maturity who show thinner, more disorganized fibre tract patterns.^7^^,^^8^ Consequently, DTI metrics can be more accurately interpreted in prepubertal children and those undergoing growth spurts compared to fully mature adolescents. The tractography findings at this stage are difficult to interpret, given that tracts can be seen in fully mature bone.^11^

The relationship between puberty, sex hormones, and growth velocity are well-known but there are still questions regarding the relationship between androgens from obesity and growth velocity. Early and mid-adolescent obese males have more advanced bone age according to GP than their normal weighted peers.^19^ A prospective study of 958 individuals found that the difference between overweight individuals and their normal weighted peers was dependent on the anatomical site.^20^ There was a significant increased odds ratio for the growth plate in the distal radius and distal tibia to be fully mature for overweight or obese individuals compared to their peers. In females there was also a significantly increased odds ratio in the distal femur and calcaneus which was not seen in males. The distribution of early growth plate maturation pattern that can be correlated to weight-related increased axial load but rather implicate the hormonal function of adipose tissue. The increase of adipose tissue seems to be a catalyst for the onset of puberty, but studies have contradictory results regarding final height.^21^^,^^22^ In conclusion, different growth plates mature at various stages of puberty and the hormonal influence on growth plate activity and closure varies from one growth plate to another. Further research with DTI to see how the activity in the growth plate varies depending on the anatomical site and age of an individual.

## Growth hormone

**Figure 5.**
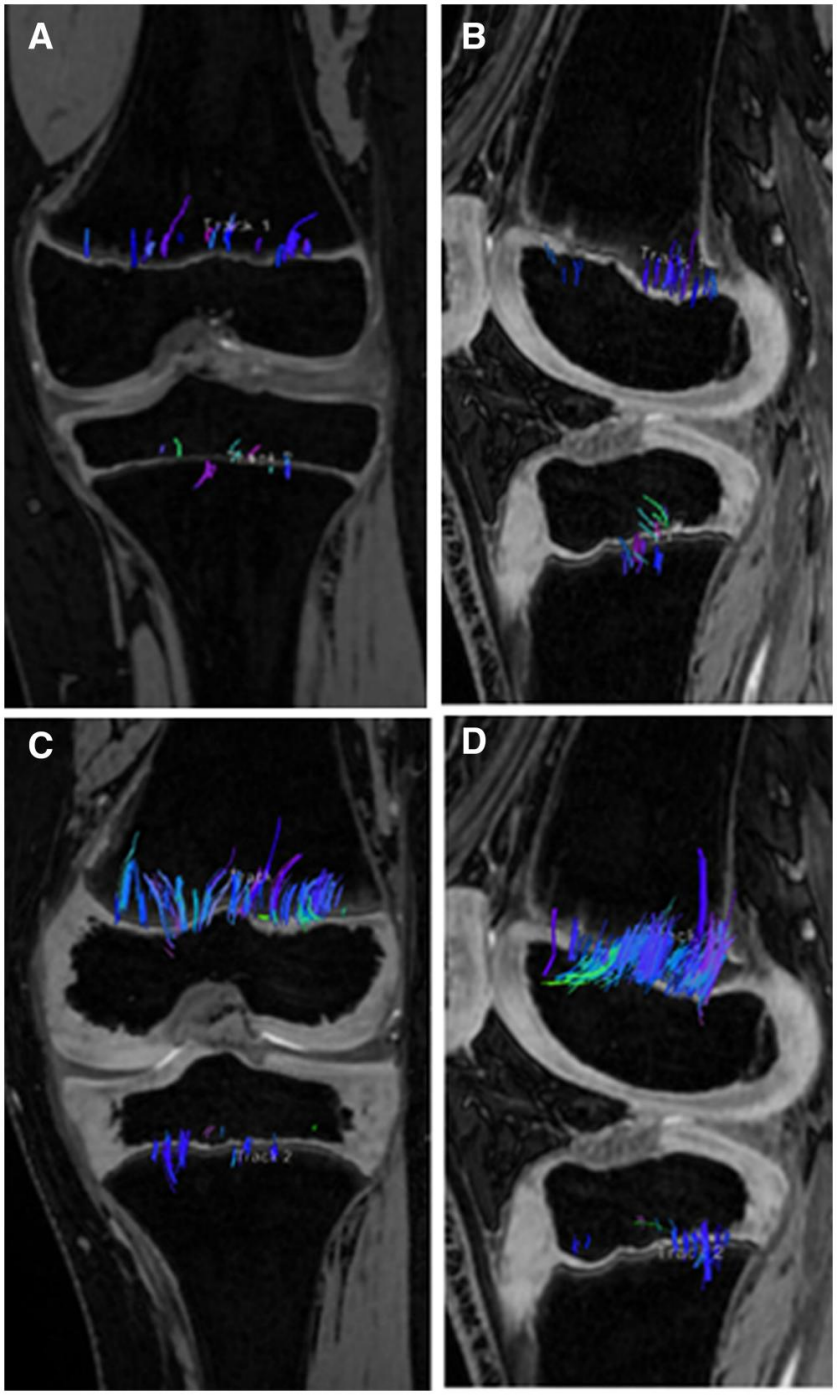
Growth hormone. Fibre tracts overlaid on conventional MR clinical coronal (A) and sagittal (B) images in a 12-year-old with a delayed bone age of 7 years old due to underlying growth hormone deficiency. MR-DTI was performed before growth hormone therapy. Fibre tracts overlaid on conventional MR clinical coronal (C) and sagittal (D) images in the same subject after 4 months of growth hormone therapy. Visible increase in fibre tract quantity and length in both femur and tibia.

The preferred treatment for children with growth hormone deficiency and short stature is growth hormone therapy.^23^ This therapy is quite expensive, and the response varies from good to none. DTI shows a snapshot of the current growth velocity and could therefore be used to evaluate the efficacy of the growth hormone therapy much faster than current methods. DTI should also be able to provide more qualitative information about the microstructure of the growth plate in addition to growth plate velocity. A good response should include more parallel tracts with an increase of tract volume whereas non-responders will show the same pattern as before treatment (Figure 5).^6^ These findings are of interest from a cost benefit perspective. First, a more sensitive detection of response enables endocrinologists to tailor the growth hormone therapy to an individual and to find the non-responders quicker. Second, it reduces the treatment cost for this patient group and the risks of unwanted side-effects related to growth hormone therapy. This theory has been tested in neuroblastoma survivors under growth hormone therapy with significant difference between the responders and non-responders.^6^

## Chemo- and radiotherapy

Several treatments have a negative effect on the growth plate. There are many known late effects related to various oncologic treatments including delayed puberty, growth failure that results in short stature of various degree.^24^ Cis-retinoic acid has a known impact on the growth plate causing reduced thickness and asymmetrical chondrocyte structure that results in advanced bone age and premature closure of the growth plates.^25^ High-risk neuroblastoma survivors showed delayed sexual maturation as well as reduced height and weight, but not BMI scores, compared to their matched peers.^6^ The neuroblastoma survivors had a reduced growth velocity of approximately 2.06 cm/year compared to their matched controls. Further, these patients presented reduced tract length, volume, and density as well as reduced FA-value (*P* < .01-.03).

A point of consideration is that a reduction of final height in neuroblastoma patients can be attributed to other treatments they have undergone, for example radiation-induced damage to the growth plates.^24^ The radiation to bone has a known cytotoxic effect on the growth plate. DTI may detect early growth plate abnormalities correlated with short stature and help identify subjects that should be considered for growth hormone therapy.

## Trauma

**Figure 6.**
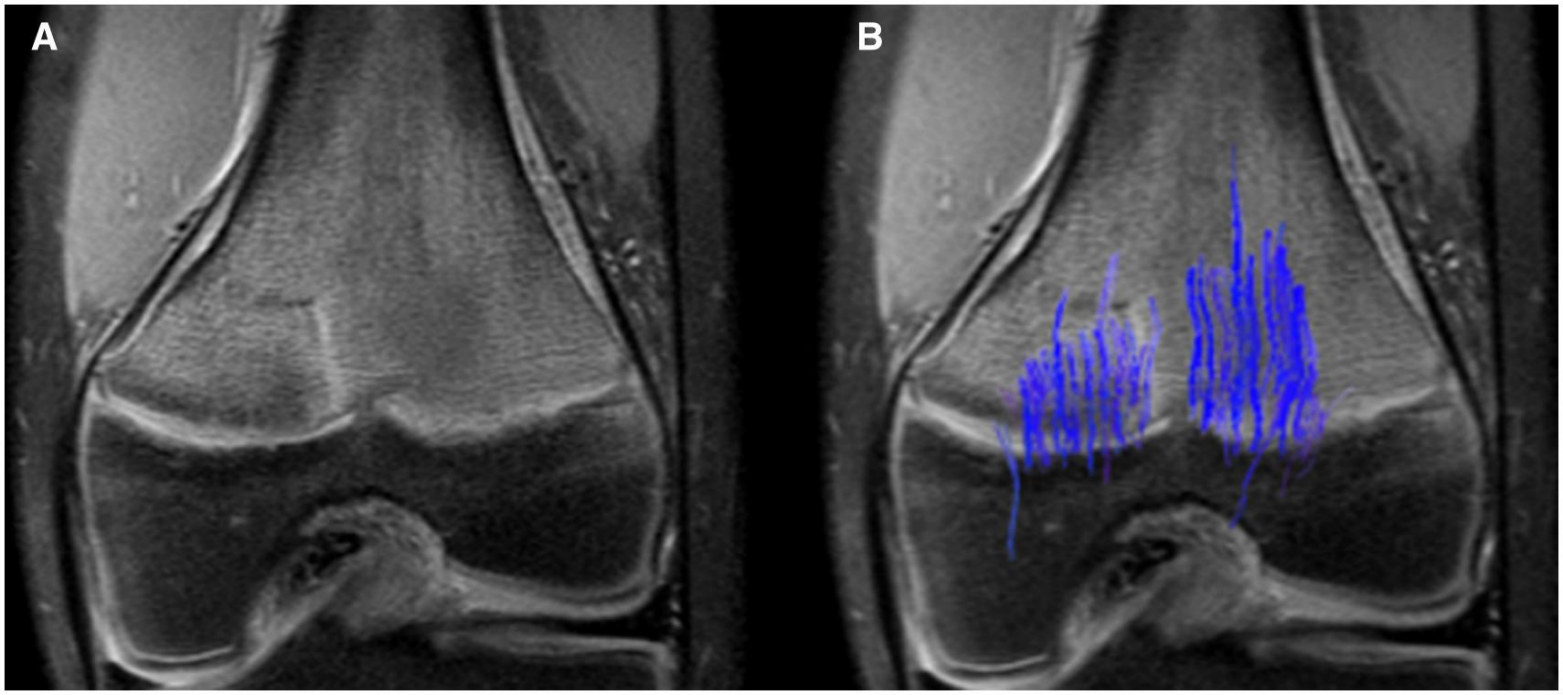
Salter Harris fracture. (A, B) Coronal proton-density image of a Salter-Harris 2 fracture in a 12-year-old-male. The fracture is in the medial portion of the growth plate with absent tracts at the fracture and decreased tract length in the surrounding area to the fracture. Courtesy of Santos et al.^13^

Animal studies have shown that both DWI^26^^,^^27^ and DTI can be used to monitor traumatic injuries that affect the growth plate.^28^ A fracture that involves the growth plate is known as a Salter-Harris fracture and can be assessed with MRI including the surrounding soft tissues.^29^ A potential complication of these fractures is bone-bridging that in turn can cause deformity and shortening, leading to leg-length discrepancies. MRI and especially DTI appears to be useful to evaluate in greater detail how the growth plate is affected by the fracture and the potential long-term effects of the growth plate (Figure 6).^13^^,^^26^

The immature skeletal system is also prone to injuries related to chronic, repetitive trauma. This stress-related trauma is dependent on biomechanics and usually sport-specific.^30^

**Figure 7.**
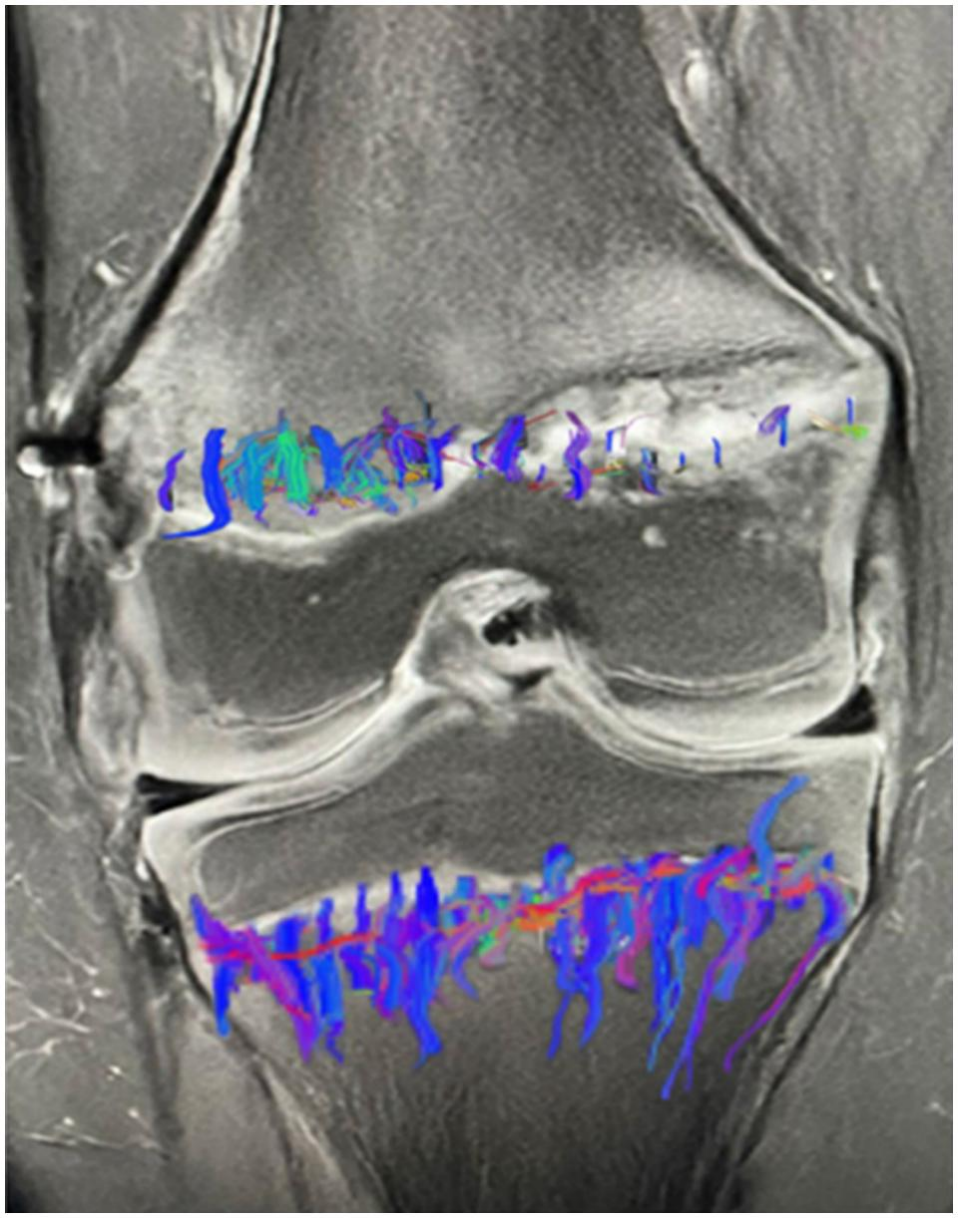
Diffusion tensor imaging of an 11-year-old boy with chronic repetitive injury to the medial portion of the growth plate. The fibre tracts are fewer and less organized than in the tibia. Courtesy of Santos et al.^13^

Repetitive trauma and chronic stress may result in a widening of the growth plate which increases the risk of injury. One of the most common sites of trauma is the wrist in gymnasts, with a widening of the growth plate especially at the volar side in symptomatic individuals.^31^ Although the data are very preliminary and the impact of DTI in these individuals is still unclear, the DTI metrics should provide insight into the microstructure of the growth plate regarding extracellular matrix through FA, MD, and RD values. In addition, the altered microstructure of the growth plate should also affect the tractography with reduced number of tracts and tract volume that might not have a symmetrical distribution (Figure 7).

## Future

Diffusion tensor imaging may prove useful in the evaluation of other pathologies involving the growing skeleton. For example, medications given to children and adolescents with neoplasms with have an inhibitory effect on the growth plate, including glucocorticoids, cis-retinoic acid, and doxorubicin. Retrospective studies, like the study of cis-retinoic acid in neuroblastoma survivors, as well as prospective animal studies are of interest to gain further insight on how much and how quickly a drug affects the growth plate but also whether the effect is irreversible.^6^ Early detection of reduced activity can be of value to ensure that an individual does not risk a reduced final height.

Early evaluation of response to growth hormone therapy is also of great interest. Medical disorders with short stature range from growth hormone sensitive to growth hormone resistant.^23^ Growth hormone-resistant patients are only diagnosable after a year of failed response, during which children receive growth hormone at a great cost and with risks of complications, but without therapeutic benefit. There is great variety within each these two patient groups and it would be very beneficial if DTI can detect early the effects of growth hormone therapy Growth hormone resistance is associated with chronic liver disease, for example Alagille syndrome, and standard growth hormone replacement has little effect on serum IGF-1.^32^ Growth hormone resistance in paediatric chronic liver disease begins early, and it is of great interest to detect it and to quantify the effect of treatments to reduce short stature. In addition, it would be of great interest to compare the effect of growth hormone therapy at different location to ensure an increase of bone length without negative side effects like acromegaly, carpal tunnel syndrome, etc.

Another area of interest is how physical activity affects the growth plate. Repetitive trauma is known to cause injuries to the growth plate. Early detection of these injuries may prevent their becoming chronic as well as understand why some individuals get chronic injuries to the growth plate while others do not.

Superimposed images are a strong tool for clinicians to understand the additional information provided by more functional imaging by linking information about function and anatomy, for example PET-MR or PET-CT. The superimposition of tractography on an MRI image will probably give the clinician additional information in a more digestible form. For instance, with leg length discrepancies difference of activity between two growth plates is evident with DTI, which may also predict how much the length difference will increase over time, months before it is detected on conventional radiographs.

## Challenges for the future

MRI-DTI of the growth plate needs to be robust, reproducible, and easily obtainable to be useful in a clinical setting. The sequence must be fast but still have a high tractography resolution (provided by the number of directions) and be sensitive to differences in diffusivity (provided by an adequate *b-value)* to be applicable in a clinical setting. Artefact elimination and reduction of noise by deep learning are tools that will provide better image quality and facilitate implementation of MRI-DTI in clinical setting. Another issue to overcome is the time-consuming and user-dependent post-processing with manual segmentation that is user-dependent and time-consuming. The development of automated segmentation tools through convolutional neural network will be of great interest in this field of research.

## Conclusion

Diffusion tensor imaging is a complement to traditional MR images that gives information regarding the microstructure of the growth plate. The diffusion metrics and tractography give us information about the maturity of the growth plate and its activity, which in turn provides information about growth velocity. The tract volume is related to growth velocity and not chronological age and the number of tracts as well as their organization can indicate area of injury.
